# The Case for GNMT as a Biomarker and a Therapeutic Target in Pancreatic Cancer

**DOI:** 10.3390/ph14030209

**Published:** 2021-03-03

**Authors:** Zachary Heinzman, Connor Schmidt, Marek K. Sliwinski, Nalin C. W. Goonesekere

**Affiliations:** 1 Department of Chemistry and Biochemistry, University of Northern Iowa, Cedar Falls, IA 50614, USA; zachary-heinzman@uiowa.edu (Z.H.); schmicaq@kcumb.edu (C.S.); 2 Department of Biology, University of Northern Iowa, Cedar Falls, IA 50614, USA; marek.k.sliwinski@uni.edu

**Keywords:** pancreatic cancer, biomarker, GNMT, 1,2,3,4,6-penta-*O*-galloyl-β-d-glucopyranoside

## Abstract

The high mortality rate for pancreatic cancer (PC) is due to the lack of specific symptoms at early tumor stages and a high biological aggressiveness. Reliable biomarkers and new therapeutic targets would help to improve outlook in PC. In this study, we analyzed the expression of GNMT in a panel of pancreatic cancer cell lines and in early-stage paired patient tissue samples (normal and diseased) by quantitative reverse transcription-PCR (qRT-PCR). We also investigated the effect of 1,2,3,4,6-penta-*O*-galloyl-β-d-glucopyranoside (PGG) as a therapeutic agent for PC. We find that GNMT is markedly downregulated (*p* < 0.05), in a majority of PC cell lines. Similar results are observed in early-stage patient tissue samples, where GNMT expression can be reduced by a 100-fold or more. We also show that PGG is a strong inhibitor of PC cell proliferation, with an IC_50_ value of 12 ng/mL, and PGG upregulates GNMT expression in a dose-dependent manner. In conclusion, our data show that GNMT has promise as a biomarker and as a therapeutic target for PC.

## 1. Introduction

Pancreatic cancer (PC) is a highly lethal cancer with poor diagnosis and dismal prognosis, with a 5 year survival rate of approximately 10%, and is the 4th leading cause of cancer-related deaths [1]. In nearly 95% of PC patients, there is neither an associated family history nor specific symptoms at the early stages of PC, when the disease can be effectively treated by surgical resection [2]. Early detection is projected to increase survival by 30–40% [3]. Recent data have suggested that the timeframe from the initiation of the pancreatic tumor to the development of metastasis could be up to two decades [4,5], suggesting a broad window of opportunity for early detection of PC.

Diagnosis of PC is usually based on radiology (CT and MRI) or invasive techniques such as ultrasound endoscopy (EUS) which are inconvenient for patients and show no superiority over existing biomarkers [6]. The biomarker CA19-9 [7], a sialylated Lewis antigen present in glycosphingolipids and glycoproteins, is the only PC biomarker approved by the US Food and Drug Administration (FDA). However, its use is discouraged for primary diagnosis of PC because of its poor sensitivity (60–70%) and specificity (70–85%) [8]. As such, CA19-9 is used clinically to monitor PC response to therapy, but its utility for screening and risk assessment is limited. In a similar vein, targeted therapies have had poor success in clinical trials of patients with PC [9] and several phase 3 clinical trials have failed [10], underscoring the need for new therapeutic targets [11]. This paucity of targeted therapies for PC has been highlighted [12]. As a consequence, there has been renewed attention on tumor-suppressor genes (TSGs) despite their reputation of being harder to “drug”.

Previously, our analysis of upregulated genes in PC [13] was able to uncover several novel candidate cancer genes and biomarkers, including CTHRC1, AHNAK2 and EPPK1, which had no previously known association with pancreatic cancer. Subsequently, AHNAK2 has been used successfully as a classifier of PC [14,15]. One of the candidate tumor-suppressor genes that we uncovered more recently [16] was GNMT, a methyltransferase that is associated with the methylation potential of the cell. GNMT expression is significantly downregulated in hepatocellular carcinoma (HCC), and GNMT knockout mice developed HCC [17], while overexpression of GNMT prevented liver cancer cell proliferation [18]. However, contrary to its role in HCC, GNMT has been shown to play a role in promoting prostate cancer cell growth and contributes to the progression of prostate cancer [19].

In this study, we have determined the expression level of GNMT in a panel of seven pancreatic cancer cell lines, and seven high-quality paired early-stage tumor tissue from PC patients. We have also evaluated the impact of a potent small molecular inhibitor of hepatocellular carcinoma, 1,2,3,4,6-penta-*O*-galloyl-β-d-glucopyranoside, which has previously been shown to act in part by upregulating the expression of GNMT [20]. We also report the analysis of Copy Number Alteration (CNA) data and patient survival data for GNMT in the TCGA PC dataset (https://www.cancer.gov/tcga (accessed on 1 August 2020)) and the UTSW dataset [21]. Taken together, our results strongly suggest that GNMT is a tumor-suppressor gene in PC, with potential to act as a diagnostic and prognostic biomarker.

## 2. Results and Discussion

### 2.1. Evidence for GNMT as a Diagnostic Biomarker for PC

**Figure 1:**
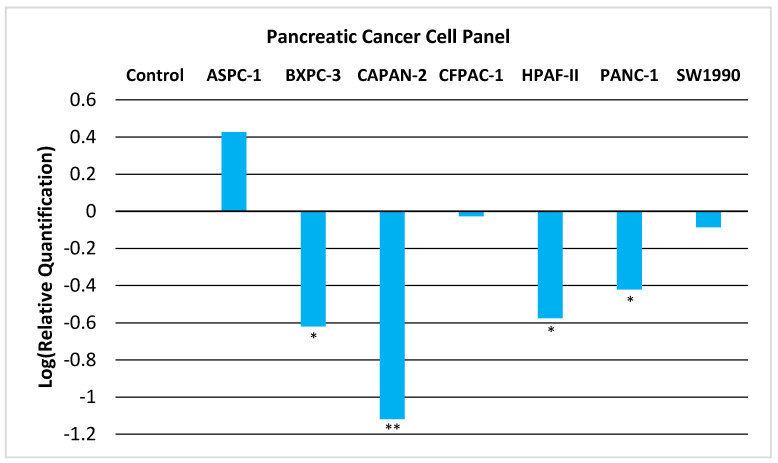
Relative Quantification (RQ) of the expression of GNMT in a Pancreatic Cancer Cell Panel by qRT-PCR. The expression of GNMT was determined and normalized to GAPDH. The Relative Quantification of the control cell line (H6c7) was set to 1 (log (RQ) = 0). Negative values for log_10_(RQ) indicate downregulation of GNMT. Significance from control, * *p* < 0.05; ** *p* < 0.005 (unpaired t-tests; *n* = 3).

We now provide experimental evidence that GNMT is downregulated in PC and thus could act as a tumor-suppressor gene. We examined the expression of GNMT in a panel of seven PC cell lines. As shown in Figure 1, GNMT is significantly downregulated (*p* < 0.05) in a majority of PC cell lines.

**Figure 2:**
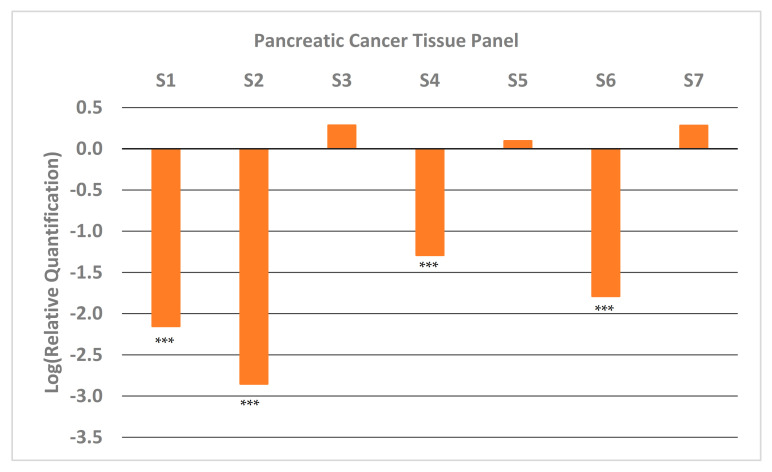
Relative Quantification (RQ) of the expression of GNMT in early-stage pancreatic cancer tumor samples from patients. Each sample was paired (PC and adjacent normal tissue) and expression of GNMT was determined and normalized to GAPDH. The expression in the normal tissue (Relative Quantification) was set to 1, and thus the Log (Relative Quantification) is 0. Significance from control, *** *p* < 0.001 (unpaired t-tests; *n* = 3).

We also looked at the expression of GNMT in paired samples (tumor and adjacent normal pancreatic tissue) of high-quality (Table S1) early-stage tumor tissue that were available at the University of Iowa Tissue Procurement Core Facility. The results from qRT-PCR (Figure 2) show that approximately half the samples have very low expression of GNMT (~100-fold less). These results are consistent with results obtained from PC cell lines (Figure 1), and illustrate the potential of GNMT as a diagnostic biomarker for PC.

Although the potential role of diagnostic biomarkers of cancer is constantly evolving, relevance and clinical application in pancreatic cancer are still limited [22]. One likely reason is the heterogenous nature of the disease, which has been classified into several tumor subtypes [23,24,25] and even two distinct stromal types [26]. In this scenario, it is likely that a panel of biomarkers will be needed to obtain the necessary metrics of sensitivity and selectivity. Indeed, in the case of CA 19.9, the only FDA approved biomarker for PC (for monitoring the progression and therapeutic response), its performance as a diagnostic biomarker for PC can be improved when used in conjunction with other candidate biomarkers [27,28,29]. A recent study [30] of a five-plasma metabolite panel of candidate biomarkers exhibited 66.7% sensitivity at 95.0% specificity. While miRNA may also be dysregulated in diseased states, the diagnostic panel of miRNA described by Schultz et al. [31] showed a sensitivity of 85%, and a specificity of 85%, and was not superior to CA19-9. These results highlight the need for identification of new biomarkers, in order to have a clinical impact through early diagnosis of PC.

### 2.2. Evidence for GNMT as a Prognostic Biomarker for PC

**Figure 3:**
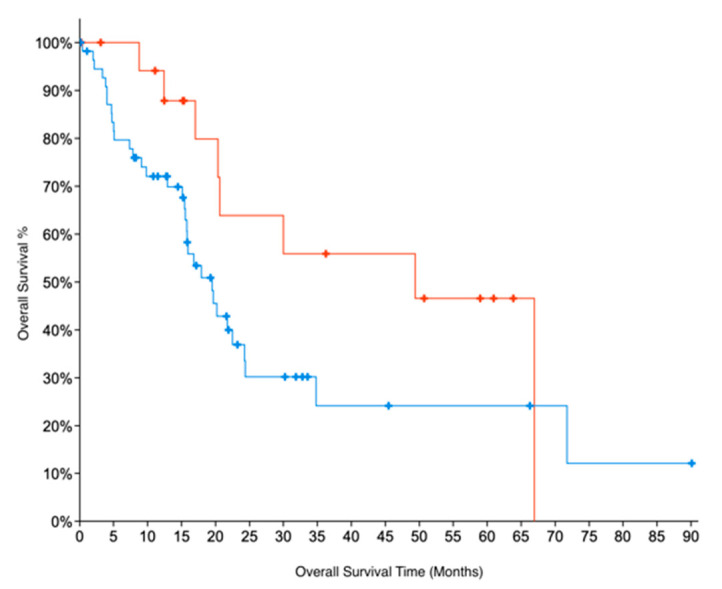
Kaplan–Meier survival curves for patients in Grade 1 (early-stage, well-differentiated) PC. Absence of deletions in GNMT (Red curve) is associated with better Overall Survival prognosis (log-rank test *p*-value = 0.08), when compared to (all) PC patients with deletions in GNMT (Blue curve).

Indirect evidence for GNMT expression can be obtained by looking at copy number alteration (CNA) data. We examined CNA data from two datasets for PC, TCGA and UTSW [21] which were accessed through cBioportal [32]. The CNA data show that 31% of samples in the TCGA dataset and 29% of samples in the UTSW dataset have deletions in GNMT. These results are also consistent with GNMT being downregulated in PC. For TCGA data, for which patient survival data are available, we further compared the overall survival of patients with deletions in GNMT. Patients with no deletions in Grade 1 (early-stage, well-differentiated) PC had twice the median months overall survival (49 months) compared to G1 patients with deletions in GNMT (22 months). The results were not significant, due to the low number of samples. Similarly, G1 samples with no deletions in GNMT had a median months overall survival that was more than double (49 vs 20 months) compared to all samples where GNMT is deleted (*p*-value = 0.08, Figure 3). These results indicate the promise of GNMT as a prognostic biomarker for PC.

### 2.3. PGG Inhibits PC Cell Proliferation, and Upregulates GNMT Expression

**Figure 4:**
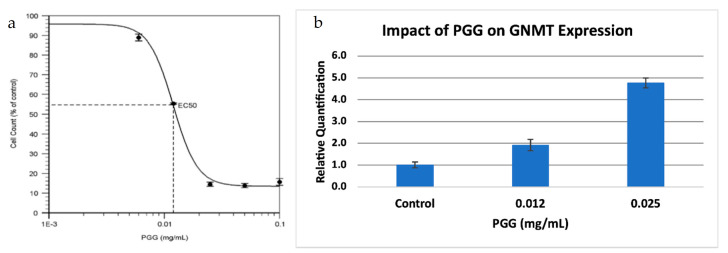
Treatment of a pancreatic cancer cell line with PGG. (**a**) Dose-response viability curve for BxPC-3 treated with PGG. The EC_50_ value of PGG for BxPC-3 is 12 ng/mL. Results are the mean ± SD (*n* = 3). (**b**). Relative Quantification (RQ) of the expression of GNMT in BxPC-3 treated with PGG, by qRT-PCR. The expression of GNMT was determined and normalized to GAPDH. The Relative Quantification of the control (0.00 mg/mL PGG) was set to 1. The cells were treated with PGG 24 h after seeding, and harvested after 72 h. Results are mean ± SD (*n* = 3).

GNMT is also a known tumor-suppressor gene in hepatocellular carcinoma (HCC). GNMT expression is significantly downregulated in HCC, and GNMT knockout mice developed HCC [17], while overexpression of GNMT prevented liver cancer cell proliferation [18]. PGG (1,2,3,4,6-penta-*O*-galloyl-β-d-glucopyranoside) is a compound that has been shown to have potent anti-HCC properties, and it has been demonstrated that the effects of PGG are linked to the upregulation of GNMT [20]. Since we also observed a downregulation of GNMT in PC, we decided to investigate the effect of PGG on PC, using a PC cell line (BxPC-3). We found that PGG was a strong inhibitor of PC cell proliferation, with an IC_50_ value similar to what was observed for HCC (Figure 4a). Further, PGG upregulated the expression of GNMT in a dose-dependent manner (Figure 4b) suggesting that PGG inhibits cell proliferation through a similar mode of action in both HCC and PC.

## 3. Materials and Methods

### 3.1. Cell Culture and Culture Conditions

Seven human pancreatic cancer cell lines and one immortalized non-tumorigenic pancreatic epithelial cell line (H6c7) were chosen for this study. The PC cell lines AsPC-1, BxPC-3, Capan-2, CFPAC-1, HPAF-II, PANC-1, and SW 1990 were obtained from the American Tissue Culture Collection (Manassas, VA, USA). Cell culture medium was as recommended by vendor. The cell line H6c7 was obtained from Dr. Ming-Sound Tsao (University of Toronto, Toronto, Ontario, Canada) and maintained in keratinocyte serum-free medium supplemented with 0.2 ng/mL epidermal growth factor and 30 μg/mL bovine pituitary extract (Invitrogen).

### 3.2. Quantitative Reverse Transcription-PCR (qRT-PCR) in Pancreatic Cancer Cell Lines

The mRNA levels of GNMT and the endogenous gene, GAPDH, were analyzed by qRT-PCR for the seven PC cell lines and control. mRNA expression data (https://portals.broadinstitute.org/ccle (accessed on 15 February 2021)) for several endogenous genes, pertaining to the seven PC cell lines, are given in Table S2. Total RNA isolation was performed using QIAshredder and the RNEasy Mini kit (Qiagen, Germantown, MD, USA). RNA quality and quantity were measured by a Nanodrop spectrophotometer (Thermo Fisher, Rochester, MN USA). One microgram of total RNA was used for reverse transcriptase (RT) reactions (20 μL total volume) to synthesize cDNA, and was carried out using the iScript cDNA Synthesis kit (Bio-Rad, Des Plaines, IL, USA). Quantitative PCR was performed in 20 μL reactions with 200 nM of each primer, iTaq Universal SYBR Green Supermix (Biorad, Bio-Rad, Des Plaines, IL, USA) and 1 μL of cDNA template using the QuantStudio 3 Real-time PCR system (Applied Biosystems, Waltham, MA, USA). Primers used were: GNMT forward 5′-ACTGGATGACTCTGGACAA-3′ and reverse 5′-CAGGGGTGCTAAGCAGTTGG-3′; GAPDH forward 5′-CCATGTTCGTCATGGGTGTG-3′ and reverse 5′-CAGGGGTGCTAAGCAGTTGG-3′. Primers were synthesized by Integrated DNA Technologies (Iowa City, IA, USA).

### 3.3. Isolation of RNA from PC Tumor Tissue

Seven paired early-stage PC tumor samples (mass ranges around 30 mg) were obtained from the University of Iowa Hospitals and Clinics. Total RNA isolation was conducted on these samples following a procedure using the Qiagen RNeasy Micro Kit as recommended by the manufacturer, with some modifications22. These modifications were necessitated due to the significant presence of RNases in the pancreas and tumor samples.

First, in a cold room at 4 °C, 900 μL of a solution containing 1 mL Buffer RLT to 50 μL of ß-Mercaptoethanol (RLT:ME) was pipetted into the 2 mL centrifuge tubes containing the tissue samples. Tissue homogenization was then conducted using a tissue homogenizer (Qiagen TissueRuptor II) set on low for 10 s and whose probes had been previously sterilized with a 70% ethanol solution and allowed to dry. This mixture was transferred to a plastic, round-bottomed test tube with an approximate size of 1.25 cm by 14 cm. The centrifuge tube was washed with another 900 μL of RLT:ME solution, and the solution was then transferred to the plastic test tube. Following this, tissue disruption was carried out using the tissue homogenizer set at full speed for 1 min on the RLT:ME tissue sample mixture. In order to achieve complete disruption, the probe was moved in a circular, up and down type pattern during all tissue disruption events.

The sample was placed on ice and, outside the cold room, transferred to a 2 mL microcentrifuge tube and centrifuged at 13,200 rpm for 3 min. Next, the supernatant was transferred, in aliquots of 900 μL, to two new 2 mL microcentrifuge tubes. 900 μL of 70% ethanol was added to each sample and mixed by pipetting. Next, 700 μL of the sample was then added to a RNeasy Spin Column and centrifuged for 15 s at 10,000 rpm. The flow-through was discarded and another 700 μL of the sample was placed into the spin column and centrifuged using the same settings as before. Finally, the flow-through was discarded, the remaining volume of sample was run through the spin column using the previous settings, and the flow-through of this final run was discarded.

After running the entire volume of sample through the spin column, 700 μL of Buffer RW1 was added to it, centrifuged for 15 s at 10,000 rpm, and the flow-through was discarded. Next, 500 μL of Buffer RPE was added to the spin column and the tube was centrifuged for 15 s at 10,000 rpm. The flow-through was discarded. Following this, an additional 500 μL of Buffer RPE was added to the spin column, centrifuged for 2 min at 10,000 rpm, and the flow-through was discarded. The spin column was again centrifuged for 1 min at 13,200 rpm to ensure complete drying of the column and was then immediately placed into a 1.5 mL microcentrifuge collection tube. 30 μL of RNase-free water was added to the spin column membrane and centrifuged for 1 min at 10,000 rpm. Immediately following RNA isolation, total RNA yields were quantified by UV Spectroscopy using a Nanodrop spectrophotometer (Thermo Fisher). Analysis of gene expression by qRT-PCR was performed as described before.

### 3.4. Determination of the EC50 Value of PGG

The PPG solution was made by initially diluting the compound in solid form to a concentration of 5× in a 1:1 ethanol to water solution which was subsequently diluted to a final volume containing 10% ethanol. 300,000 cells were added to each well in a 6-well tissue culture plate with 1.5 mL of medium. After 24 h, the medium was replaced with a new medium containing PGG (0.1, 0.05, 0.025, 0.012, and 0.006 mg/mL along with a control well containing no PGG). After 72 h, the wells were washed with PBS and the remaining cells were counted using a Bio-Rad TC10 Automated Cell Counter.

## 4. Conclusions

In this paper, we examined the status of GNMT, a gene identified in our previous studies as a putative tumor-suppressor gene and biomarker for PC. We looked at the expression levels of GNMT in a panel of seven pancreatic cancer cell lines, and confirmed that GNMT is significantly downregulated in a majority of these cell lines. Importantly, we were also able to show that GNMT is downregulated by 100-fold or more in approximately half of the high-quality early-stage paired tumor tissue samples obtained from the University of Iowa Tissue Procurement Core Facility. These results support a role for GNMT as a diagnostic biomarker for PC.

We also obtained indirect evidence for GNMT expression levels from an analysis of copy number alteration (CNA) data from two studies (TCGA and UTSW), which showed significant gene deletions for GNMT. In addition, patient survival data, which is available for the TCGA PC dataset, show that GNMT has promise as a prognostic biomarker of PC.

Finally, we provide preliminary evidence that the compound PGG (1,2,3,4,6-penta-O-galloyl-β-D-glucopyranoside) shows promise as a therapeutic agent for PC. It causes cell death in pancreatic cancer cells, with an EC_50_ value of 12 ng/mL. The EC_50_ value is similar to the cell toxicity observed for hepatocellular carcinoma [20]. Further, we show that PGG upregulates GNMT expression in a PC cell line, suggesting a role for GNMT as a tumor suppressor. According to copy number alteration data from TCGA and UTSW datasets, approximately 1/3 of PC samples have a deletion in GNMT.

If our preliminary findings are confirmed, we have a new actionable therapeutic target for PC in GNMT, that can reach a significant proportion of the patient population.

## Data Availability

Data is contained within the article or Supplementary Materials.

## Supplementary Materials

The following are available online at https://www.mdpi.com/1424-8247/14/3/209/s1, Table S1: Clinicopathological characteristics of patient tumor tissue samples. Table S2: The coefficients of variation calculated from mRNA expression (RNAseq) data (https://portals.broadinstitute.org/ccle (accessed on 15 February 2021)) for the seven PC cell lines used in this study, for some common endogenous genes.

## References

[1] Siegel R.L., Miller K.D., Jemal A. (2020). Cancer statistics. CA Cancer J. Clin..

[2] Chakraborty S., Baine M.J., Sasson A.R., Batra S.K. (2011). Current status of molecular markers for early detection of sporadic pancreatic cancer. Biochim. Biophys. Acta.

[3] Rahib L., Smith B.D., Aizenberg R., Rosenzweig A.B., Fleshman J.M., Matrisian L.M. (2014). Projecting cancer incidence and deaths to 2030: The unexpected burden of thyroid, liver, and pancreas cancers in the United States. Cancer Res..

[4] Nai Q., Luo H., Zhang P., Hossain M.A., Gu P., Sidhom I.W., Mathew T., Islam M., Yousif A.M., Sen S. (2015). How early can pancreatic cancer be recognized? A case report and review of the literature. Case Rep. Oncol..

[5] Yachida S., Jones S., Bozic I., Antal T., Leary R., Fu B., Kamiyama M., Hruban R.H., Eshleman J.R., Nowak M.A. (2010). Distant metastasis occurs late during the genetic evolution of pancreatic cancer. Nature.

[6] DaVee T., Coronel E., Papafragkakis C., Thaiudom S., Lanke G., Chakinala R.C., González G.M.N., Bhutani M.S., Ross W.A., Weston B.R. (2018). Pancreatic cancer screening in high-risk individuals with germline genetic mutations. Gastrointest Endosc.

[7] Goonetilleke K.S., Siriwardena A.K. (2007). Systematic review of carbohydrate antigen (CA 19-9) as a biochemical marker in the diagnosis of pancreatic cancer. Eur. J. Surg. Oncol..

[8] Caputo D., Caracciolo G. (2020). Nanoparticle-enabled blood tests for early detection of pancreatic ductal adenocarcinoma. Cancer Lett..

[9] Middleton G., Palmer D.H., Greenhalf W., Ghaneh P., Jackson R., Cox T., Evans A., Shaw V.E., Wadsley J., Valle J.W. (2017). Vandetanib plus gemcitabine versus placebo plus gemcitabine in locally advanced or metastatic pancreatic carcinoma (ViP): A prospective, randomised, double-blind, multicentre phase 2 trial. Lancet Oncol..

[10] Mizrahi J.D., Surana R., Valle J.W., Shroff R.T. (2020). Pancreatic cancer. Lancet.

[11] Kleeff J., Michl P. (2017). Targeted therapy of pancreatic cancer: Biomarkers are needed. Lancet Oncol..

[12] Amanam I., Chung V. (2018). Targeted Therapies for Pancreatic Cancer. Cancers.

[13] Goonesekere N.C., Wang X., Ludwig L., Guda C. (2014). A meta analysis of pancreatic microarray datasets yields new targets as cancer genes and biomarkers. PLoS ONE.

[14] Bhasin M.K., Ndebele K., Bucur O., Yee E.U., Otu H.H., Plati J., Bullock A., Gu X., Castan E., Zhang P. (2016). Meta-analysis of transcriptome data identifies a novel 5-gene pancreatic adenocarcinoma classifier. Oncotarget.

[15] Almeida P.P., Cardoso C.P., de Freitas L.M. (2020). PDAC-ANN: An artificial neural network to predict pancreatic ductal adenocarcinoma based on gene expression. BMC Cancer.

[16] Goonesekere N.C.W., Andersen W., Smith A., Wang X. (2018). Identification of genes highly downregulated in pancreatic cancer through a meta-analysis of microarray datasets: Implications for discovery of novel tumor-suppressor genes and therapeutic targets. J. Cancer Res. Clin. Oncol..

[17] Martínez-Chantar M.L., Vázquez-Chantada M., Ariz U., Martínez N., Varela M., Luka Z., Capdevila A., Rodríguez J., Aransay A.M., Matthiesen R. (2008). Loss of the glycine N-methyltransferase gene leads to steatosis and hepatocellular carcinoma in mice. Hepatology.

[18] Yen C.H., Lu Y.C., Li C.H., Lee C.M., Chen C.Y., Cheng M.Y., Huang S.F., Chen K.F., Cheng A.L., Liao L.Y. (2012). Functional characterization of glycine N-methyltransferase and its interactive protein DEPDC6/DEPTOR in hepatocellular carcinoma. Mol. Med..

[19] Song Y.H., Shiota M., Kuroiwa K., Naito S., Oda Y. (2011). The important role of glycine N-methyltransferase in the carcinogenesis and progression of prostate cancer. Mod. Pathol..

[20] Kant R., Yen C.H., Lu C.K., Lin Y.C., Li J.H., Chen Y.M. (2016). Identification of 1,2,3,4,6-Penta-O-galloyl-β-d-glucopyranoside as a Glycine N-Methyltransferase Enhancer by High-Throughput Screening of Natural Products Inhibits Hepatocellular Carcinoma. Int. J. Mol. Sci.

[21] Witkiewicz A.K., McMillan E.A., Balaji U., Baek G., Lin W.C., Mansour J., Mollaee M., Wagner K.U., Koduru P., Yopp A. (2015). Whole-exome sequencing of pancreatic cancer defines genetic diversity and therapeutic targets. Nat. Commun..

[22] Zhang L., Sanagapalli S., Stoita A. (2018). Challenges in diagnosis of pancreatic cancer. World J. Gastroenterol..

[23] Chan-Seng-Yue M., Kim J.C., Wilson G.W., Ng K., Figueroa E.F., O’Kane G.M., Connor A.A., Denroche R.E., Grant R.C., McLeod J. (2020). Transcription phenotypes of pancreatic cancer are driven by genomic events during tumor evolution. Nat. Genet..

[24] Bailey P., Chang D.K., Nones K., Johns A.L., Patch A.M., Gingras M.C., Miller D.K., Christ A.N., Bruxner T.J., Quinn M.C. (2016). Genomic analyses identify molecular subtypes of pancreatic cancer. Nature.

[25] Collisson E.A., Sadanandam A., Olson P., Gibb W.J., Truitt M., Gu S., Cooc J., Weinkle J., Kim G.E., Jakkula L. (2011). Subtypes of pancreatic ductal adenocarcinoma and their differing responses to therapy. Nat. Med..

[26] Moffitt R.A., Marayati R., Flate E.L., Volmar K.E., Loeza S.G.H., Hoadley K.A., Rashid N.U., Williams L.A., Eaton S.C., Chung A.H. (2015). Virtual microdissection identifies distinct tumor- and stroma-specific subtypes of pancreatic ductal adenocarcinoma. Nat. Genet..

[27] Makawita S., Dimitromanolakis A., Soosaipillai A., Soleas I., Chan A., Gallinger S., Haun R.S., Blasutig I.M., Diamandis E.P. (2013). Validation of four candidate pancreatic cancer serological biomarkers that improve the performance of CA19. BMC Cancer.

[28] Brand R.E., Nolen B.M., Zeh H.J., Allen P.J., Eloubeidi M.A., Goldberg M., Elton E., Arnoletti J.P., Christein J.D., Vickers S.M. (2011). Serum biomarker panels for the detection of pancreatic cancer. Clin. Cancer Res..

[29] Park H.D., Kang E.S., Kim J.W., Lee K.T., Lee K.H., Park Y.S., Park J.O., Lee J., Heo J.S., Choi S.H. (2012). Serum CA19-9, cathepsin D, and matrix metalloproteinase-7 as a diagnostic panel for pancreatic ductal adenocarcinoma. Proteomics.

[30] Fahrmann J.F., Bantis L.E., Capello M., Scelo G., Dennison J.B., Patel N., Murage E., Vykoukal J., Kundnani D.L., Foretova L. (2018). A Plasma-Derived Protein-Metabolite Multiplexed Panel for Early-Stage Pancreatic Cancer. J. Natl. Cancer Inst..

[31] Schultz N.A., Dehlendorff C., Jensen B.V., Bjerregaard J.K., Nielsen K.R., Bojesen S.E., Calatayud D., Nielsen S.E., Yilmaz M., Holländer N.H. (2014). MicroRNA biomarkers in whole blood for detection of pancreatic cancer. JAMA.

[32] Cerami E., Gao J., Dogrusoz U., Gross B.E., Sumer S.O., Aksoy B.A., Jacobsen A., Byrne C.J., Heuer M.L., Larsson E. (2012). The cBio cancer genomics portal: An open platform for exploring multidimensional cancer genomics data. Cancer Discov..

