# Supplementary figures and images for: The Case for GNMT as a Biomarker and a Therapeutic Target in Pancreatic Cancer
Source: Pharmaceuticals (Basel). 2021 Mar 3;14(3):209. doi: 10.3390/ph14030209 (PMC7998508; doi:10.3390/ph14030209)

Table SI: Clinicopathological characteristics of patient tumor tissue samples


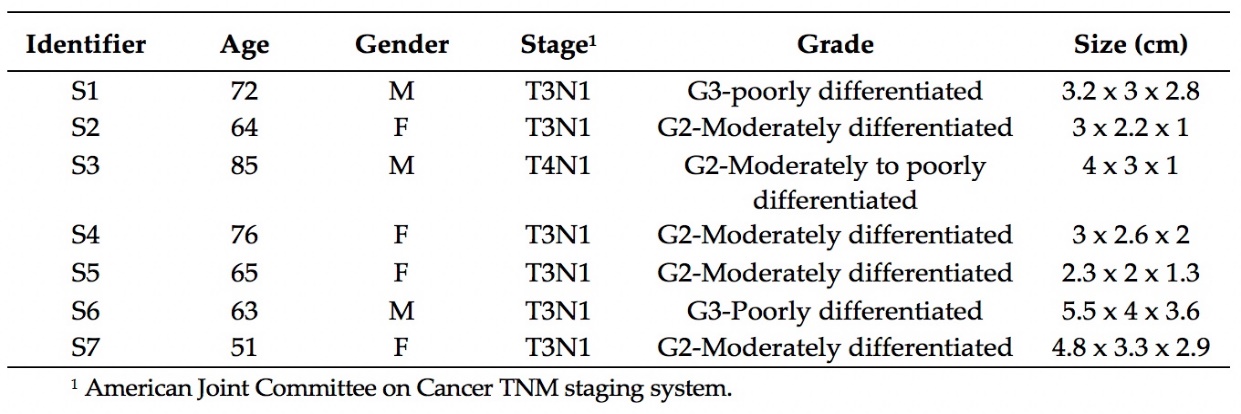

Supplement: Supplementary file 1 [file pharmaceuticals-14-00209-s001.zip › pharmaceuticals-1074144-suppl/Table S1.docx]
